# Horizontal gene transfer plays a major role in the pathological convergence of *Xanthomonas* lineages on common bean

**DOI:** 10.1186/s12864-018-4975-4

**Published:** 2018-08-13

**Authors:** Nicolas W. G. Chen, Laurana Serres-Giardi, Mylène Ruh, Martial Briand, Sophie Bonneau, Armelle Darrasse, Valérie Barbe, Lionel Gagnevin, Ralf Koebnik, Marie-Agnès Jacques

**Affiliations:** 1 IRHS, INRA, AGROCAMPUS OUEST, Université d’Angers, SFR4207 QUASAV, 42, rue Georges Morel, 49071 Beaucouzé, France; 2 CEA/DSV/IG/Genoscope, 2 rue Gaston Crémieux, BP5706, 91057 Evry, France; 3 CIRAD, UMR PVBMT, F-97410 Saint-Pierre, La Réunion France; 4 IRD, CIRAD, Université de Montpellier, IPME, Montpellier, France

**Keywords:** *Xanthomonas*, Common bean, TAL effectors, Host adaptation, Horizontal gene transfer

## Abstract

**Background:**

Host specialization is a hallmark of numerous plant pathogens including bacteria, fungi, oomycetes and viruses. Yet, the molecular and evolutionary bases of host specificity are poorly understood. In some cases, pathological convergence is observed for individuals belonging to distant phylogenetic clades. This is the case for *Xanthomonas* strains responsible for common bacterial blight of bean, spread across four genetic lineages. All the strains from these four lineages converged for pathogenicity on common bean, implying possible gene convergences and/or sharing of a common arsenal of genes conferring the ability to infect common bean.

**Results:**

To search for genes involved in common bean specificity, we used a combination of whole-genome analyses without a priori, including a genome scan based on *k*-mer search. Analysis of 72 genomes from a collection of *Xanthomonas* pathovars unveiled 115 genes bearing DNA sequences specific to strains responsible for common bacterial blight, including 20 genes located on a plasmid. Of these 115 genes, 88 were involved in successive events of horizontal gene transfers among the four genetic lineages, and 44 contained nonsynonymous polymorphisms unique to the causal agents of common bacterial blight.

**Conclusions:**

Our study revealed that host specificity of common bacterial blight agents is associated with a combination of horizontal transfers of genes, and highlights the role of plasmids in these horizontal transfers.

**Electronic supplementary material:**

The online version of this article (10.1186/s12864-018-4975-4) contains supplementary material, which is available to authorized users.

## Background

In nature, most pathogens are generalists, meaning that they are able to infect multiple hosts, while other pathogens are specialists, meaning that they are highly adapted to a single or few host species [1]. For plant pathogens, adaptation to a specific host plant is a complex process possibly involving diverse molecular determinants and leading to host specificity [2, 3]. Understanding the molecular basis of host specificity can provide new insights into the evolution and ecology of specialist pathogens, and their potential to shift species and to infect new hosts. Bacteria from the genus *Xanthomonas* infect at least 392 plant species including important crops and ornamentals [4]. Yet, each individual strain is able to infect only one or few plant species. Strains able to cause the same symptoms on the same host range are grouped into pathovars [5]. Although our understanding of the molecular basis of host specificity is still limited, chemotactic sensors, adhesins and type III effectors emerge as key determinants for shaping host specificity in *Xanthomonas* [6–8]. Chemotactic sensors enable the bacteria to detect attractant or repellent molecules and trigger flagellar motility towards entry sites of the host plant, while adhesins allow the attachment on the host plant surface and biofilm formation, and type III effectors are delivered into the plant cells where they can have different functions including providing pathogen-associated molecular pattern triggered immunity (PTI).

The *Xanthomonas axonopodis* species complex sensu Vauterin [9, 10] groups more than 30 pathovars infecting a wide range of plants including economically important crops and ornamentals, such as *Citrus*, *Anthurium* and *Dieffenbachia* species, as well as pepper, cassava, cotton, mango, soybean, and common bean. Based on repetitive-sequence-based Polymerase Chain Reaction (rep-PCR) fingerprints, *X. axonopodis* has been subdivided into six subclusters named 9.1 to 9.6 [11]. More recently, this species complex has been split into the four species *X. citri*, *X. euvesicatoria*, *X. phaseoli* and *X. axonopodis* [12, 13]. Common bacterial blight of bean (CBB) is the most devastating bacterial disease infecting common bean (*Phaseolus vulgaris* L.). CBB occurs everywhere where common bean is cultivated and may cause up to 75% yield loss in the most severe cases [14, 15]. *Xanthomonas* strains responsible for CBB are distributed across four different genetic lineages [16]. The fuscous lineage (fuscans) and the non-fuscous lineages 2 (NF2) and 3 (NF3) belong to *X. citri* pv. *fuscans* while the non-fuscous lineage 1 (NF1) belongs to *X. phaseoli* pv. *phaseoli* [9, 11, 12]. Pathological convergence between the NF1 and fuscans lineages is associated with horizontal gene transfers (HGT) involving dozens of genes [17]. Horizontal transfer of genes encoding Transcription Activator-Like (TAL) type III effectors was also observed between the four lineages of CBB agents [18]. In particular, all strains from the four genetic lineages display an allele of the *tal23A* gene, suggesting that this gene is important for *Xanthomonas* adaptation to common bean.

In order to search for *Xanthomonas* genes putatively involved in the adaptation leading to common bean specificity in *Xanthomonas*, we have generated the whole genome sequences of 17 *X. citri* pv. *fuscans* and *X. phaseoli* pv. *phaseoli* strains. A combination of approaches including a comparison between the phylogeny of genes and the phylogeny of organisms, a parsimony approach to infer gene gains and losses, and a genome-wide search for specific *k*-mers, was used to search for genes presenting common characteristics unique to strains belonging to the four bean-pathogenic lineages of *X. citri* pv. *fuscans* and *X. phaseoli* pv. *phaseoli*.

## Results

### Genome sequencing and phylogeny

**Fig. 1:**
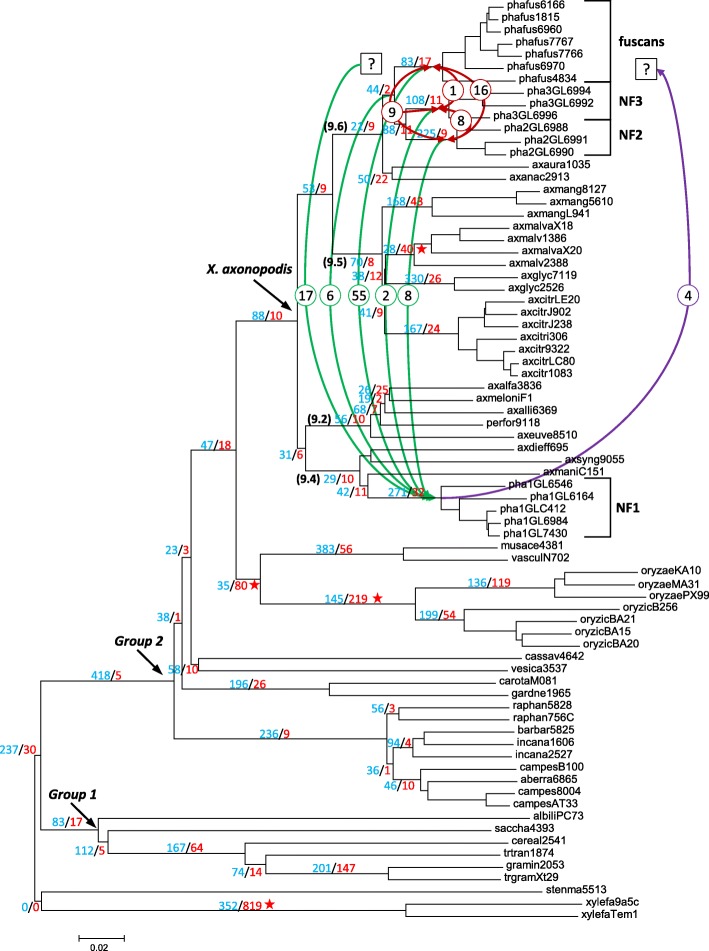
Phylogeny of *Xanthomonas* strains used in this study. Phylogeny of *Xanthomonas* strains used in this study with indication of gene gains and losses. The phylogenetic tree is based on whole genome analysis using CVTree [66] with default parameters. Strain aliases are described in Table 1. *Stenotrophomonas* and *Xylella* genomes have been used as outgroups. *Xanthomonas* main phylogenetic groups 1 and 2 [24] and the *X. axonopodis* species complex [9, 10] are indicated by arrows. Groups 9.2, 9.4, 9.5 and 9.6 [11] are indicated in brackets. Fuscans, NF2, NF3 and NF1 refer to the four genetic lineages of strains responsible for CBB. A parsimony approach was performed to infer gene gains (blue) and losses (red) at levels higher than the pathovar rank, and numbers are displayed at each branch. Red stars highlight cases where gene loss was greater that gene gain. Curved arrows represent horizontal gene transfers (HGT) retrieved by Ks analysis on alignments of 115 candidate genes for bean specificity, with HGT from *X. citri* pv. *fuscans* to *X. phaseoli* pv. *phaseoli* in green, HGT from *X. phaseoli* pv. *phaseoli* to *X. citri* pv. *fuscans* in purple, and HGT between *X. citri* pv. *fuscans* lineages in red. Numbers in circles correspond to the numbers of candidate genes involved for each HGT. Question marks indicate events for which the origin or end of the HGT was not precise enough to assign any particular lineage

In order to obtain genomic data representative of the diversity of *Xanthomonas* strains responsible for CBB, we produced whole genome sequences for 17 strains from the four genetic lineages of *X. citri* pv. *fuscans* and *X. phaseoli* pv. *phaseoli* that affect beans. In addition, we sequenced two strains of *X. citri* pv. *mangiferaeindicae*, one strain of *X. citri* pv. *anacardii*, three strains of *X. oryzae* pv. *oryzicola*, and used 51 other publically available *Xanthomonas* genomes for a total of 72 whole genome sequences (Table 1). *Stenotrophomonas maltophilia* strain R551–3 and *Xylella fastidiosa* strains 9a5c and Temecula1 were used as outgroups for further analyses [19–21]. Annotation revealed from 3209 to 5405 coding sequences (CDS) per *Xanthomonas* genome (Additional file 1). Among *Xanthomonas* strains responsible for CBB, chromosome size ranged from 4,957,446 bp in strain CFBP1815 to 5,517,999 bp in strain CFBP6992, with an average GC content ranging from 64.3 to 64.9%. The phylogeny of strains was assessed based on the amino acid sequences of all annotated CDS using CVTree (Fig. 1). The overall topology of this tree was congruent with previous *Xanthomonas* phylogenies [11, 12, 22]. As described previously, strains responsible for CBB are distributed into four distinct genetic lineages belonging to two different species, *X. citri* and *X. phaseoli* [12, 13, 16, 23].

**Table 1:** Informations on the sequenced strains used in this study

Identifier	Species/Pathovar	Strain	Host of isolation	Country and date of isolation	Contigs	Total size (bp)	GC%	Accession number	Reference
stenma5513	*Stenotrophomonas maltophilia*	R551-3	*Populus trichocarpa*	na	1	4,573,969	66.29	GCA_000020665.1	Taghavi et al., 2008 [21]
albiliPC73	*X. albilineans*	GPE PC73	*Saccharum* spp. cv. H63-1418.	Guadeloupe (France), 2003	1	3,768,695	62.97	GCA_000087965.1	Pieretti et al., 2009 [77]
aberra6865	*X. campestris* pv. *campestris*	CFBP6865	*Brassica oleracea* var. *capitata.*	Australia, 1975	4	5,169,518	64.93	Unpublished	Noël, comm. pers
barbar5825	*X. campestris* pv. *barbareae*	CFBP5825R	*Barbarea vulgaris*	USA, 1939	2	5,055,186	65.09	ATNQ00000000	Roux et al., 2015 [78]
campes8004	*X. campestris* pv. *campestris*	8004	*Brassica oleracea* var. *botrytis.*	United Kingdom, 1958	1	5,148,708	64.95	GCA_000012105.1	Qian et al., 2005 [79]
campesAT33	*X. campestris* pv. *campestris*	ATCC 33913	*Brassica oleracea* var. *gemmifera*	United Kingdom, 1957	1	5,076,188	65.06	GCA_000007145.1	da Silva et al., 2002 [80]
campesB100	*X. campestris* pv. *campestris*	B100	na	na	1	5,079,002	65.04	GCA_000070605.1	Vorhölter et al., 2008 [81]
incana2527	*X. campestris* pv. *incanae*	CFBP2527R	*Matthiola incana*	USA, 1950	1	4,926,205	65.12	ATNO00000000	Roux et al., 2015 [78]
incana1606	*X. campestris* pv. *incanae*	CFBP1606R	*Matthiola incana*	France, 1974	1	4,967,651	65.17	ATNN00000000	Roux et al., 2015 [78]
musace4381	*X. campestris* pv. *musacearum*	NCPPB4381	*Musa* sp.	Uganda, 2005	115	4,810,038	61.96	ACHT00000000	Studholme et al., 2010 [82]
raphan5828	*X. campestris* pv. *raphani*	CFBP5828R	*Raphanus sativus*	USA, na	2	4,912,709	65.35	ATNP00000000	Roux et al., 2015 [78]
raphan756C	*X. campestris* pv. *raphani*	756C	*Brassica oleracea* var. *capitata*	east asia, na	1	4,941,214	65.28	GCA_000221965.1	Bogdanove et al., 2011 [83]
cassav4642	*X. cassavae*	CFBP4642	*Manihot esculenta*	Malawi, 1951	7	5,278,192	65.22	GCA_000454545.1	Bolot et al., 2013 [84]
axanac2913	*X. citri* pv. *anacardii*	CFBP2913	*Mangifera indica*	Brazil, na	1	5,203,496	64.57	CP024057	this study
axaura1035	*X. citri* pv. *aurantifolii*	ICPB10535	*Citrus aurantiifolia*	Brazil, na	351	5,060,896	64.81	ACPY00000000	Moreira et al., 2010 [85]
axcitr1083	*X. citri* pv. *citri*	FDC1083	*Citrus reticulata*	Brazil, 1980	1	5,218,314	64.72	CCVZ01000000	Gordon et al., 2015 [86]
axcitrJ902	*X. citri* pv. *citri*	JF90-2	*Citrus aurantiifolia*	Oman, 1986	1	5,251,225	64.66	CCWA01000000	Gordon et al., 2015 [86]
axcitrJ238	*X. citri* pv. *citri*	JJ238-24	*Citrus aurantiifolia*	Thailand, 1989	1	5,282,622	64.78	CCVX01000000	Gordon et al., 2015 [86]
axcitrLC80	*X. citri* pv. *citri*	LC80	*Citrus reticulata* x *C. sinensis*	Mali, 2006	1	5,229,127	64.7	CCWJ01000000	Gordon et al., 2015 [86]
axcitrLE20	*X. citri* pv. *citri*	LE20-1	*Citrus aurantiifolia*	Ethiopia, 2008	1	5,309,240	64.71	CCWK01000000	Gordon et al., 2015 [86]
axcitr9322	*X. citri* pv. *citri*	LMG9322	*Citrus aurantiifolia*	Florida, 1986	1	5,194,131	64.75	CCVY01000000	Gordon et al., 2015 [86]
axcitri306	*X. citri* pv. *citri*	306	na	na	1	5,175,554	64.77	GCA_000007165.1	da Silva et al., 2002 [80]
phafus1815	*X. citri* pv. *fuscans* (fuscans)	CFBP1815	*Phaseolus* sp	Greece, 1978	1	4,957,446	64.82	GCA_900234415	this study
phafus4834	*X. citri* pv. *fuscans* (fuscans)	4834-R	*Phaseolus vulgaris* cv. Michelet	France, 1998	1	4,981,995	64.81	FO681494	Darrasse et al., 2013 [26]
phafus6166	*X. citri* pv. *fuscans* (fuscans)	CFBP6166	*Phaseolus vulgaris*	South Africa, 1963	1	4,987,587	64.8	GCA_900234455	this study
phafus6960	*X. citri* pv. *fuscans* (fuscans)	CFBP6960	*Phaseolus vulgaris*	Reunion island, France, 2000	1	4,992,131	64.79	GCA_900234515	this study
phafus6970	*X. citri* pv. *fuscans* (fuscans)	CFBP6970	*Phaseolus* sp	USA, 1990	1	5,006,656	64.9	GCA_900234505	this study
pha2GL6988	*X. citri* pv. *fuscans* (NF2)	CFBP6988	*Phaseolus vulgaris* cv. marla	Reunion island, France, 2000	1	5,132,433	64.64	Deposited	this study
pha2GL6990	*X. citri* pv. *fuscans* (NF2)	CFBP6990	*Phaseolus vulgaris* cv. marla	Reunion island, France, 2000	1	5,124,653	64.63	GCA_900234485	this study
pha2GL6991	*X. citri* pv. *fuscans* (NF2)	CFBP6991	*Phaseolus vulgaris* cv. marla	Reunion island, France, 2000	1	5,342,565	64.35	GCA_900234525	this study
pha3GL6992	*X. citri* pv. *fuscans* (NF3)	CFBP6992	*Phaseolus vulgaris* cv. marla	Reunion island, France, 2000	1	5,517,999	64.48	GCA_900234495	this study
pha3GL6994	*X. citri* pv. *fuscans* (NF3)	CFBP6994	*Phaseolus vulgaris*	Tanzania, 1990	1	5,250,266	64.57	GCA_900234565	this study
pha3GL6996	*X. citri* pv. *fuscans* (NF3)	CFBP6996	*Phaseolus vulgaris* cv. marla	Reunion island, France, 2000	4	5,095,420	64.72	AVET00000000	this study
phafus7766	*X. citri* pv. *fuscans* (fuscans)	CFBP7766	*Phaseolus vulgaris*	Cameroon, 2009	1	5,185,891	64.63	GCA_900234475	this study
phafus7767	*X. citri* pv. *fuscans* (fuscans)	CFBP7767	*Phaseolus vulgaris*	Cameroon, 2009	1	5,107,388	64.7	GCA_900234465	this study
axglyc2526	*X. citri* pv. *glycines*	CFBP2526	*Glycine hispida*	Sudan, 1956	4	5,255,152	64.63	GCA_000495275.1	Darrasse et al., 2013 [26]
axglyc7119	*X. citri* pv. *glycines*	CFBP7119	*Glycine max*	Brazil, 1981	4	5,521,783	64.39	GCA_000488895.1	Darrasse et al., 2013 [26]
axmalv1386	*X. citri* pv. *malvacearum*	GSPB1386	na	na	127	4,991,411	64.7	GCA_000309905.1	Hainan University
axmalv2388	*X. citri* pv. *malvacearum*	GSPB2388	na	na	61	5,127,016	64.53	GCA_000309925.1	Hainan University
axmalvaX18	*X. citri* pv. *malvacearum*	X18	*Gossypium* spp.	Burkina Faso, 1980s	4	4,993,692	64.7	ATMA00000000	Cunnac et al., 2013 [87]
axmalvaX20	*X. citri* pv. *malvacearum*	X20	*Gossypium* spp.	Burkina Faso, 1980s	4	5,219,648	64.49	ATMB00000000	Cunnac et al., 2013 [87]
axmang5610	*X. citri* pv. *mangiferaeindicae*	LG56-10	*Mangifera indica*	na	4	5,281,952	64.56	PEBY00000000	this study
axmang8127	*X. citri* pv. *mangiferaeindicae*	LG81-27	*Mangifera indica*	na	6	5,209,594	64.68	PEBZ00000000	this study
axmangL941	*X. citri* pv. *mangiferaeindicae*	LMG941	*Mangifera indica*	India, 1957	195	5,144,323	64.84	CAHO01000001	Midha et al., 2012 [88]
axalfa3836	*X. euvesicatoria* pv. *alfalfae*	CFBP3836	*Medicago sativa*	Sudan, na	6	5,081,438	64.74	GCA_000488955.1	Jacques et al., 2013 [3, 22]
axalli6369	*X. euvesicatoria* pv. *allii*	CFBP6369	*Allium cepa*	Reunion island, France, 1996	3	5,427,488	64.36	GCA_000730305.1	Gagnevin et al., 2014 [89]
axmeloniF1	*X. euvesicatoria* pv. *citrumelonis*	F1	*Citrus* sp.	FL, USA, 1984	1	4,967,469	64.91	GCA_000225915.1	Jalan et al., 2011 [90]
axeuve8510	*X. euvesicatoria* pv. *euvesicatoria*	85-10	*Capsicum annuum*	FL, USA, 1985	1	5,178,466	64.74	GCA_000009165.1	Thieme et al., 2005 [91]
perfor9118	*X. euvesicatoria* pv. *perforans*	91-118	*Lycopersicon esculentum*	FL, USA, na	291	5,296,241	65.04	AEQW00000000	Potnis et al., 2011 [92]
gardne1965	*X. gardneri*	ATCC19865	*Lycopersicon esculentum*	Yugoslavia, 1953	552	5,594,687	63.68	AEQX00000000	Potnis et al., 2011 [92]
carotaM081	*X. hortorum* pv. *carotae*	M081	*Daucus carota*	OR, USA, na	154	5,039,269	63.83	GCA_000505565.1	Kimbrel et al., 2011 [93]
oryzaeKA10	*X. oryzae* pv. *oryzae*	KACC10331	*Oryza sativa*	Korea, na	1	4,941,439	63.69	GCA_000007385.1	Lee et al., 2005 [94]
oryzaeMA31	*X. oryzae* pv. *oryzae*	MAFF 311018	*Oryza sativa*	Japan, na	1	4,940,217	63.7	GCA_000010025.1	unpublished
oryzaePX99	*X. oryzae* pv. *oryzae*	PXO99A	*Oryza sativa*	Philippines, na	1	5,240,075	63.63	GCA_000019585.1	Salzberg et al., 2008 [95]
oryzicBA15	*X. oryzae* pv. *oryzicola*	BAI15	*Oryza sativa*	Burkina Faso, 2009	1	4,315,327	64.16	GCA_002189395.1	this study
oryzicBA20	*X. oryzae* pv. *oryzicola*	BAI20	*Oryza sativa*	Burkina Faso, 2009	1	4,498,643	64.05	GCA_002189435.1	this study
oryzicBA21	*X. oryzae* pv. *oryzicola*	BAI21	*Oryza sativa*	Burkina Faso, 2009	1	4,419,870	64.07	GCA_002189465.1	this study
oryzicB256	*X. oryzae* pv. *oryzicola*	BLS256	*Oryza sativa*	Philippines, 1984	1	4,831,739	64.05	AAQN01000001	Bogdanove et al., 2011 [83]
axdieff695	*X. phaseoli* pv. *dieffenbachiae*	LMG695	*Anthurium andreanum*	Brazil, 1965	1	5,037,357	64.88	GCA_001564415.1	Robène et al., 2016 [96]
axmaniC151	*X. phaseoli* pv. *manihotis*	CIO151	*Manihot* sp.	Colombia, na	36	5,154,532	64.7	GCA_000265845.1	Rodriguez et al., 2012 [97]
pha1GLC412	*X. phaseoli* pv. *phaseoli* (NF1)	CFBP412	*Phaseolus vulgaris*	USA, na	1	5,028,351	64.94	GCA_900234435	this study
pha1GL6164	*X. phaseoli* pv. *phaseoli* (NF1)	CFBP6164	*Phaseolus vulgaris*	Romania, 1996	1	5,319,061	64.64	GCA_900234535	this study
pha1GL6546	*X. phaseoli* pv. *phaseoli* (NF1)	CFBP6546	*Phaseolus vulgaris*	USA, na	4	4,994,665	64.92	Deposited	this study
pha1GL6984	*X. phaseoli* pv. *phaseoli* (NF1)	CFBP6984	*Phaseolus vulgaris*	Reunion island, France, 2000	1	5,106,663	64.82	GCA_900234425	this study
pha1GL7430	*X. phaseoli* pv. *phaseoli* (NF1)	CFBP7430	*Phaseolus vulgaris* cv. CFO16	Iran, 2006	1	5,085,978	64.89	GCA_900234445	this study
axsyng9055	*X. phaseoli* pv. *syngonii* (NF1)	LMG9055	*Syngonium podophyllum*	na	6	5,010,905	64.82	GCA_001640215.1	Robène et al., 2016 [96]
saccha4393	*X. sacchari*	NCPPB4393	*Musa* sp.	Tanzania, 2007	470	4,955,099	69.03	AGDB00000000	Studholme et al., 2011 [98]
cereal2541	*X. translucens* pv. *cerealis*	CFBP2541	*Bromus inermis*	USA, 1941	10	4,524,870	67.37	GCA_000807145.1	Pesce et al., 2015a [99]
trgramXt29	*X. translucens* pv. *graminis*	ART-Xtg29	*Lolium multiflorum*	Switzerland, na	788	4,203,855	68.58	ANGG00000000	Wichman et al., 2013 [101]
gramin2053	*X. translucens* pv. *graminis*	CFPB2053	*Dactylis glomerata*	Switzerland, 1973	2	4,344,936	68.33	LHSI00000000	Pesce et al., 2015b [100]
trtran1874	*X. translucens* pv. *translucens*	DSM18974	*Hordeum vulgare*	USA, 1958	551	4,550,921	67.67	GCA_000331775.1	unpublished
vasculN702	*X. vasicola* pv. *vasculorum*	NCPPB702	*Saccharum officinarum*	Zimbabwe, 1959	97	5,491,457	56.86	ACHS00000000	Studholme et al., 2010 [82]
vesica3537	*X. vesicatoria*	ATCC35937	*Lycopersicon esculentum*	New Zealand, 1955	296	5,567,561	64.06	AEQV01000000	Potnis et al., 2011 [92]
xylefaTem1	*Xylella fastidiosa* subsp. *fastidiosa*	Temecula1	*Vitis vinifera*	USA, na	1	2,519,802	51.77	GCA_000007245.1	van Sluys et al., 2003 [102]
xylefa9a5c	*Xylella fastidiosa* subsp. *pauca*	9a5c	*Citrus sinensis* cv. Valencia	Brazil, 1992	1	2,679,306	52.67	GCA_000006725.1	Simpson et al., 2000 [19]

*na* not available

### Genome expansion occurred during the evolution of *Xanthomonas*

To identify the genes shared by different clades of *Xanthomonas*, we constructed an orthology matrix using OrthoMCL (Additional file 2). Based on this orthology matrix, we performed a parsimony approach to infer gene gains and losses at each branch of the phylogenetic tree (Fig. 1). We did not take into account events occurring on the most distal branches to reduce the bias due to the difference of quality between sequenced genomes. At every branch, one to several hundreds of genes were either gained or lost. A general observation was that gene gains were higher than gene losses, suggesting that genome expansion occurred during the evolution of *Xanthomonas* (Fig. 1). Only four cases of genome reduction were observed (i) at the origin of the *Xylella* genus, (ii and iii) along two consecutive branches before and after the split between the *X. oryzae* species and the *X. vasculorum* and *X. musacearum* species, and (iv) at the origin of *X. citri* pv. *malvacearum*. The largest gene gain (418) was observed at the origin of *Xanthomonas* phylogenetic group 2 as defined by Young et al. [24], while the largest gene loss (819) was observed at the origin of the *Xylella* genus. Few gene losses (9 to 32) were observed before the diversification of each of the four genetic lineages involved in CBB. Of those, the NF1 lineage was the one which gained the most genes (271) followed by the NF2 (225), NF3 (108) and fuscans (83) lineages, respectively.

### The pan and core genomes of *Xanthomonas* reveal extensive horizontal gene transfers between strains pathogenic on common bean

**Fig. 2:**
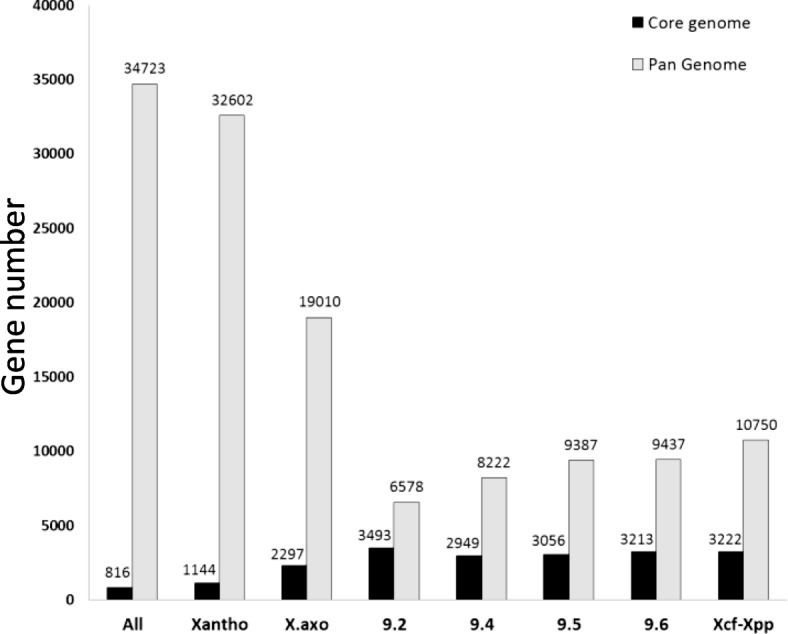
The core- and pan-genome of *Xanthomonas*. Gene numbers correspond to the number of ortholog groups retrieved for each group of strains. All: all strains used in this study (*n* = 75); Xantho: strains from the *Xanthomonas* genus (*n* = 72); X.axo: strains from the *Xanthomonas axonopodis* species complex (*n* = 44). 9.2, 9.4, 9.5, 9.6: strains belonging to rep-PCR groups 9.2 (*n* = 5), 9.4 (*n* = 8), 9.5 (*n* = 16) and 9.6 (*n* = 15), respectively, as defined in Rademaker et al. [103]. Xcf-Xpp: strains pathogenic on common bean belonging to *Xanthomonas citri* pv. *fuscans* or *Xanthomonas phaseoli* pv. *phaseoli* (*n* = 18)

Individuals that are closely related to each other typically share more orthologs than unrelated individuals. Therefore, groups of closely related individuals tend to have a smaller pan genome and a larger core genome than groups of more divergent individuals. As such, the pan and core genomes for the 72 *Xanthomonas* strains comprised 32,602 and 1144 CDS, respectively, while the pan and core genomes for the 75 strains including the outgroups comprised 34,723 and 816 CDS, respectively (Fig. 2). Similarly, each Rademaker group alone, i.e. 9.2, 9.4, 9.5 and 9.6, had a smaller pan genome (6578, 8222, 9387 and 9437 CDS, respectively) and a larger core genome (3493, 2949, 3056 and 3213 CDS, respectively) than the *X. axonopodis* species complex, which had pan and core genomes of 19,010 and 2297 CDS, respectively. Strikingly, when grouping strains responsible for CBB belonging to *X. citri* pv. *fuscans* and *X. phaseoli* pv. *phaseoli*, both the pan and core genomes (10,750 and 3222 CDS, respectively) were larger than the pan and core genomes from groups 9.4 (8222 and 2949 CDS, respectively) or 9.6 (9437 and 3213 CDS, respectively) (Fig. 2). Thus, strains responsible for CBB, although being phylogenetically diverse, had more genes in common than they had with other strains belonging to their respective clades, which was suggestive of extensive HGT among these strains. This result was reminiscent of previous comparative analyses showing that dozens of genes have been horizontally transferred between the fuscans and NF1 lineages [17].

### Strains pathogenic on common bean share 115 CDS presenting unique characteristics

To search for genes potentially involved in the convergence between *X. phaseoli* pv. *phaseoli* (i.e. the NF1 lineage) and *X. citri* pv. *fuscans* (i.e. the NF2, NF3 and fuscans lineages) to infect common bean, we performed a combination of four different analyses. First, within the OrthoMCL matrix, we searched for CDS specifically present in the genomes of CBB agents and absent from any other *Xanthomonas* genome, or present in the genomes from all *Xanthomonas* but not in the genomes of CBB agents. No CDS was retrieved by this analysis. We also searched for CDS specifically present or absent when grouping the NF1 lineage to each of the NF2, NF3, or fuscans lineages. Only one CDS was specifically retrieved in the NF1 and fuscans lineages.

Second, we used the results from the CDS gains and losses approach described above to search for genes shared by all strains from *X. phaseoli* pv. *phaseoli* and *X. citri* pv. *fuscans*, and gained in the ancestor of one pathovar or the other. This approach unveiled nine CDS shared by all strains responsible for CBB, and gained in either *X. phaseoli* pv. *phaseoli* or *X. citri* pv. *fuscans* (Table 2). We also searched for CDS shared by the NF1 and each of the NF2, NF3, or fuscans lineage and gained in at least one of these lineages. Four CDS were shared by the NF1 and NF2 lineages, or the NF1 and NF3 lineages, while three CDS were shared by the NF1 and fuscans lineages.

**Table 2:** Numbers of CDS presenting similarities among the lineages of CBB agents

Lineages studied	Presence/absence^a^	Gained^b^	Monophyletic^c^	24-mers^d^
NF1/NF2/NF3/fuscans	0	9	28	108
NF1/NF2	0	4	9	33
NF1/NF3	0	4	5	28
NF1/fuscans	1	3	105	231
Total	1	20	147	400

^a^CDS specifically present or absent in all the lineages studied compared to all other *X. axonopodis* strains

^b^CDS present in all the lineages studied and gained in a least one of these lineages

^c^CDS monophyletic for the lineages studied

^d^CDS containing 24-mers specifically present or absent in all the lineages studied compared to all other *X.axonopodis* strains

Third, we used a phylogenetic approach to search for genes for which strains from *X. citri* pv. *fuscans* and *X. phaseoli* pv. *phaseoli* formed a monophyletic group. For this, we constructed phylogenetic trees on 3202 CDS present in every *X. citri* pv. *fuscans* and *X. phaseoli* pv. *phaseoli* strain and in at least one additional strain from group 9.2 or 9.4 and one other strain from group 9.5 or 9.6. The additional strains from groups 9.2, 9.4, 9.5 and 9.6 were located inbetween *X. citri* pv. *fuscans* and *X. phaseoli* pv. *phaseoli* (Fig. 1). Thus, CDS found as monophyletic for CBB strains could be potential traces of HGT between both pathovars. This approach unveiled 28 CDS for which the four genetic lineages formed a monophyletic group, suggesting that they were horizontally transferred among these lineages (Table 2). Nine CDS were specifically monophyletic for the NF1 and NF2 lineages, five for the NF1 and NF3 lineages, and 105 for the NF1 and fuscans lineages, suggesting that most horizontal transfers occurred among the NF1 and fuscans lineages.

Finally, we used the SkIf tool [25] on the 72 *Xanthomonas* genomes to search for genes containing short 24-bp sequences (24-mers) specific to strains responsible for CBB, or alternatively genes from strains responsible for CBB lacking 24-mers present in all other strains from the *X. axonopodis* species complex. In all, we identified 108 CDS containing 24-mers either specifically present or absent from the four lineages (Table 2). Moreover, 33 CDS contained 24-mers specific for the NF1 and NF2 lineages, 28 for the NF1 and NF3 lineages and 231 for the NF1 and fuscans lineages. Similarly to the analysis based on phylogeny, this analysis based on k-mers pointed an overrepresentation of CDS with specific 24-mers shared by the NF1 and fuscans lineages compared to NF2 and NF3 lineages.

Together, these four analyses unveiled respectively 0, 9, 28, or 109 CDS presenting features unique to CBB agents. The analysis based on presence/absence seemed to be too stringent for unveiling any CDS, while the analysis based on k-mers was the most sensitive, suggesting that SkIf was an appropriate tool for finding common traits shared by phylogenetically distant strains. Most of these CDS were found redundantly by two or more analyses, for a total of 115 non-redundant CDS (Table 3). The most represented functions encoded by these 115 predicted CDS were hypothetical proteins (26 CDS), followed by membrane-related proteins (10 CDS), two-component system proteins (six CDS), putative secreted proteins (five CDS), reductases (five CDS), RNA-related proteins (five CDS), Type III secretion system-related proteins (five CDS), TonB-dependent proteins (four CDS), Type IV secretion system-related proteins (three CDS), Type VI secretion system-related proteins (three CDS), DNA-related proteins (three CDS) and transcription regulators (three CDS) (Table 3).

**Table 3:** Overview of the 115 genes putatively involved in bean specificity

Identifier^a^	Accession number^b^	Predicted function	Gene	Gained	Monophyletic	24-mers	Recombinant	HGT	Atypical GC%	Nonsynonymous sites	Aritua et al. [17]^c^
1	m00100560	TonB-dependent transporter	–	–	–	Yes	–	Yes	–		–
2	m00100580	type III effector	*avrBs2*	–	–	Yes	Yes	Yes	–	–	–
3	m00100590	hypothetical protein	–	–	–	Yes	–	Yes	–	Yes	–
4	m00101230	two compoment system sensor protein	–	–	–	Yes	–	–	–	–	–
5	m00101980	two component system protein	*glnG*	–	–	Yes	–	–	–	–	–
6	m00102200	two component system response regulator	–	–	Yes	Yes	–	Yes	–	–	Yes
7	m00104250	type III secretion system protein	*hrpF*	–	–	Yes	Yes	Yes	–	–	–
8	m00104520	type III effector	*xopA*	–	–	Yes	–	Yes	–	Yes	–
9	m00104530	lytic transglycosylase-like protein	*hpaH*	–	–	Yes	–	Yes	–	Yes	Yes
10	m00104540	hypothetical protein	–	–	–	Yes	–	Yes	–	Yes	–
11	m00104640	diguanylate cyclase	–	–	–	Yes	–	Yes	–	Yes	–
12	m00104690	4-alpha-glucaNotransferase	*malQ*	–	–	Yes	–	Yes	–	–	–
13	m00105290	threonine aldolase	–	–	–	Yes	–	Yes	–	–	–
14	m00105330	flavin reductase	–	–	Yes	Yes	–	Yes	–	Yes	–
15	m00105340	anthranilate phosphoribosyl transferase	*trpD*	–	–	Yes	–	Yes	–	–	–
16	m00105390	S-adenosylmethionine decarboxylase	*speD*	–	–	Yes	–	Yes	Yes	–	–
17	m00105750	hypothetical membrane protein	–	–	–	Yes	–	–	–	–	–
18	m00107500	TonB-dependent transporter	–	–	–	Yes	Yes	Yes	–	–	–
19	m00108560	general secretion pathway protein D	*xpsD*	–	Yes	Yes	Yes	Yes	–	Yes	–
20	m00108570	hypothetical protein	–	–	Yes	Yes	–	Yes	–	–	–
21	m00108600	TonB-dependent transporter	–	–	–	Yes	Yes	Yes	–	Yes	–
22	m00109610	TonB-dependent transporter	–	–	–	Yes	Yes	Yes	–	–	–
23	m00109620	phosphoanhydride phosphohydrolase	*appA*	–	Yes	Yes	Yes	Yes	–	Yes	–
24	m00109670	hypothetical protein	–	–	–	Yes	–	Yes	–	–	–
25	m00109990	bis(5′nucleosyl)-tetraphosphatase	*apaH*	–	–	Yes	–	–	–	–	–
26	m00110970	peptidyl-tRNA hydrolase	–	–	Yes	Yes	–	Yes	–	–	Yes
27	m00111160	50S ribosomal protein	*rplW*	–	Yes	–	–	Yes	–	–	–
28	m00112070	two-component system sensor protein	*phoR*	–	–	Yes	–	–	–	–	–
29	m00113470	xanthine dehydrogenase subunit	–	–	Yes	Yes	Yes	Yes	–	Yes	–
30	m00113490	xanthine dehydrogenase subunit	–	–	–	Yes	–	Yes	–	–	–
31	m00114150	membrane protein	–	–	Yes	–	–	Yes	–	–	Yes
32	m00114680	two component system sensor protein	–	–	–	Yes	Yes	Yes	–	–	–
33	m00116790	NAD/FAD binding protein	–	–	Yes	–	–	Yes	–	–	–
34	m00117590	acetyltransferase	–	–	–	Yes	–	Yes	–	–	–
35	m00117600	transcriptional regulator	–	–	Yes	Yes	Yes	Yes	–	Yes	–
36	m00117610	hypothetical protein	–	–	–	Yes	–	Yes	–	Yes	–
37	m00118890	hypothetical protein	–	–	Yes	–	–	Yes	–	–	–
38	m00119700	putative secreted protein	–	–	Yes	–	–	–	–	–	–
39	m00119970	phosphomethylpyrimidine kinase	*thiD*	–	–	Yes	–	Yes	–	–	Yes
40	m00121810	threonine synthase	*thrC*	–	Yes	Yes	–	Yes	–	Yes	–
41	m00121850	histidyl tRNA synthetase	*hisS*	–	–	Yes	–	Yes	–	–	–
42	m00121900	histidinol phosphatase	*hisB*	–	–	Yes	–	Yes	–	–	Yes
43	m00123210	recombination factor protein	*rarA*	–	–	Yes	–	Yes	–	–	–
44	m00126530	ethanolamine permease	*eutP*	–	Yes	Yes	–	Yes	–	–	–
45	m00126540	ethanolamine ammona-lyase	*eutB*	–	–	Yes	–	Yes	–	–	–
46	m00127140	membrane protein	–	–	–	Yes	–	Yes	–	–	–
47	m00127150	cGMP specific phosphodiesterase	–	–	Yes	Yes	–	Yes	–	Yes	–
48	m00127220	hypothetical protein	–	–	–	Yes	–	Yes	–	–	–
49	m00127250	ubiquinol-cytochrome c reductase subunit	–	–	–	Yes	–	Yes	–	–	–
50	m00127260	ubiquinol-cytochrome c reductase subunit	–	–	–	Yes	–	Yes	–	–	–
51	m00127850	1-phosphofructokinase	*fruK*	–	–	Yes	–	Yes	–	–	–
52	m00127860	PTS fructose porter IIBC component	*fruA*	–	–	Yes	–	Yes	–	–	–
53	m00130650	RNA polymerase ECF-type sigma factor	*rpoE2*	–	–	Yes	–	Yes	–	Yes	–
54	m00130660	hypothetical protein	–	–	Yes	Yes	–	Yes	–	Yes	–
55	m00130670	RNA-binding protein	–	–	–	Yes	–	Yes	–	–	Yes
56	m00130690	inner membrane protein	–	–	–	Yes	–	Yes	–	–	–
57	m00132430	DNA topoisomerase IA	–	–	–	Yes	–	–	–	Yes	–
58	m00135840	membrane fusion protein	–	–	–	Yes	–	–	–	–	–
59	m00135870	sulfite reductase (NADPH) subunit	*cysJ*	–	–	Yes	Yes	Yes	–	–	–
60	m00135880	sulfite reductase (NADPH) subunit	*cysI*	–	–	Yes	–	Yes	–	–	–
61	m00135970	transcriptional regulator protein	*cysB*	–	–	Yes	–	Yes	–	–	–
62	m00135980	siroheme synthase	*cysG*	–	Yes	Yes	–	Yes	–	–	–
63	m00136050	phosphoglycerate kinase	*pgk*	–	–	Yes	–	Yes	–	–	–
64	m00136550	hypothetical protein	–	–	–	Yes	–	–	–	–	–
65	m00138140	two-compoment system sensor protein	–	–	–	Yes	–	Yes	–	–	–
66	m00139330	dihydrolipoamide acetyltransferase	*aceF*	–	–	Yes	Yes	Yes	–	–	–
67	m00139350	DNA glycosylase	–	–	Yes	Yes	–	Yes	–	–	–
68	m00139420	membrane protein	–	–	–	Yes	–	Yes	–	Yes	Yes
69	m00139860	putative secreted protein	–	–	–	Yes	–	–	–	–	–
70	m00139930	flavodoxin protein	–	–	–	Yes	–	Yes	–	–	–
71	m00140860	peptidoglycan binding protein	*lysM*	–	Yes	–	–	Yes	–	–	–
72	m00140880	hypothetical protein	–	–	–	Yes	–	–	–	–	–
73	m00141310	PhnB like protein	–	–	–	Yes	–	Yes	–	Yes	–
74	m00141340	hypothetical protein	–	–	–	Yes	–	Yes	–	–	–
75	m00141400	hypothetical protein	–	–	–	Yes	–	Yes	–	–	–
76	m00141430	transmembrane protein	–	–	–	Yes	–	Yes	–	–	Yes
77	m00141440	hypothetical protein	–	–	Yes	Yes	–	Yes	–	–	–
78	m00141490	dipeptide epimerase	–	–	–	Yes	–	Yes	–	–	–
79	m00142140	putative glucosyltransferase	–	–	–	Yes	Yes	Yes	–	–	–
80	m00144120	type VI secretion system protein	*icmF*	–	–	Yes	Yes	Yes	–	Yes	–
81	m00144240	type VI secretion system kinase protein	*tagE*	–	–	Yes	Yes	Yes	–	–	–
82	m00144270	transmembrane sensor protein	*fecR*	–	–	Yes	–	Yes	–	–	–
83	m00144420	type VI secretion system virulence protein	*impE*	–	–	Yes	Yes	Yes	–	–	–
84	m00145330	type III effector	*xopAD*	–	–	Yes	Yes	Yes	–	Yes	–
85	m00146940	membrane protein	–	–	–	Yes	Yes	Yes	–	–	–
86	m00146970	LysR family transcriptional regulator	–	–	–	Yes	–	Yes	–	–	–
87	m00146980	short chain dehydrogenase	–	–	–	Yes	–	Yes	–	–	–
88	m00146990	RNA 2′-phosphotransferase family protein	–	–	–	Yes	–	Yes	–	Yes	–
89	m00147020	hypothetical protein	–	–	Yes	Yes	–	Yes	–	–	–
90	m00147060	esterase family protein	–	–	–	Yes	–	Yes	–	–	–
91	m00147090	putative secreted protein	–	–	–	Yes	–	Yes	–	–	–
92	m00147100	putative secreted protein	–	–	–	Yes	–	Yes	–	Yes	–
93	m00147120	ribosomal pseudouridine synthase	–	–	Yes	Yes	–	Yes	–	Yes	–
94	m00147130	hypothetical protein	–	–	–	Yes	–	Yes	–	Yes	–
95	m00147140	putative secreted protein	–	–	–	Yes	–	Yes	–	Yes	–
96	m00200020	plasmid partitioning protein	–	–	Yes	Yes	–	na	–	Yes	–
97	m00200060	hypothetical protein	–	Yes	–	Yes	–	na	–	Yes	–
98	m00200080	hypothetical protein	–	Yes	–	Yes	–	na	–	Yes	–
99	m00200130	hypothetical protein	–	–	–	Yes	–	Yes	–	Yes	–
100	m00200150	hypothetical protein	–	Yes	–	Yes	–	na	–	Yes	–
101	m00200160	hypothetical protein	–	–	–	Yes	–	na	Yes	Yes	–
102	m00200170	hypothetical protein	–	Yes	–	–	–	na	–	Yes	–
103	m00200190	hypothetical protein	–	–	–	Yes	–	na	–	Yes	–
104	m00200260	hypothetical protein	–	–	–	Yes	–	Yes	–	Yes	–
105	m00200790	transposase	*tnpA*	–	–	Yes	–	Yes	–	Yes	–
106	m00200820	resolvase	*tnpR*	–	–	Yes	–	Yes	–	–	–
107	m00200880	DNA topoisomerase IA	–	–	–	Yes	–	na	–	Yes	–
108	m00200980	type IV secretion system protein	*trbI*	Yes	Yes	Yes	–	na	–	Yes	–
109	m00201120	membrane protein	–	–	–	Yes	–	–	–	–	–
110	m00201220	type IV conjugal transfer protein	*traG*	–	–	Yes	–	na	–	Yes	–
111	m00201230	hypothetical protein	–	–	–	Yes	–	na	–	Yes	–
112	m00201250	lytic transglycolase	–	Yes	Yes	Yes	–	na	–	Yes	–
113	m00201260	type IV conjugal transfer protein	*traF*	Yes	–	Yes	–	na	–	Yes	–
114	m00201270	hypothetical protein	–	Yes	Yes	Yes	–	na	–	Yes	–
115	m00201290	hypothetical protein	–	Yes	Yes	Yes	–	na	–	Yes	–
Total	115	–	*–*	9	28	108	18	88	2	44	9

– not found in corresponding analysis

*na* not applicable due to the absence of corresponding CDS in outgroup

**Fig. 3:**
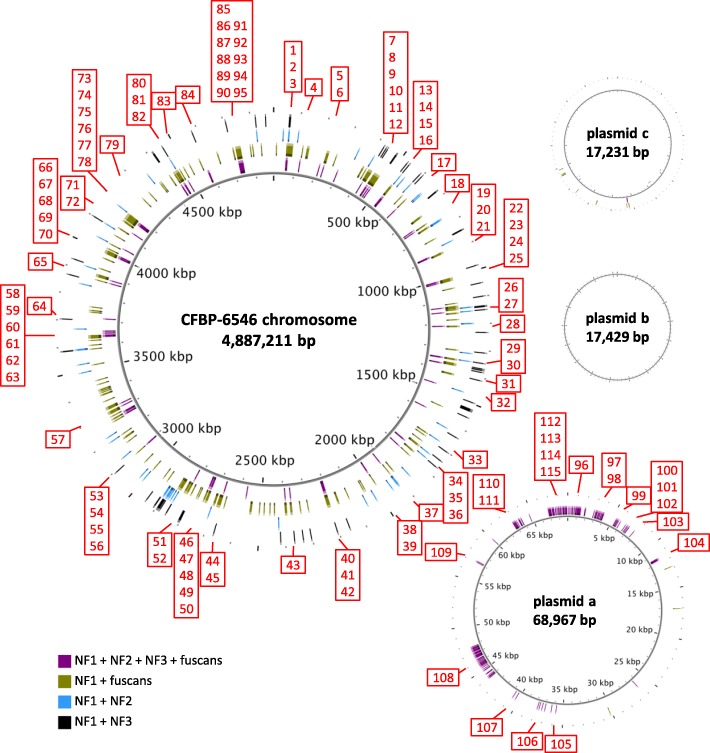
Mapping of the 24-mers specific for strains pathogenic on common bean. The innermost rings represent the reference chromosome or plasmids with associated coordinates. Colored lines represent 24-mers specifically retrieved in *X. citri* pv. *fuscans* and *X. phaseoli* pv. *phaseoli* strains (purple), or in the NF1 plus fuscans lineages (green), or in the NF1 plus NF2 lineages (blue), or in the NF1 plus NF3 lineages (black). Red numbers correspond to the identifiers of the 115 genes listed in Table 3

^a^identifiers correponding to the numbers shown in Fig. 3

^b^accession numbers for strain CFBP6456

^c^genes with 100% identity over 95% of their length according to Aritua et al. [17]

We hypothesized that the CDS potentially involved in the specific adaptation to common bean should bear nonsynonymous polymorphisms specific to *Xanthomonas* strains pathogenic on common bean. Analysis of the alignments for the 115 candidate CDS highlighted 44 CDS with nonsynonymous sites retrieved exclusively in *X. citri* pv. *fuscans* and *X. phaseoli* pv. *phaseoli* (Table 3). More than one third of these CDS (16/44) encoded hypothetical proteins. Among the other CDS, three encoded type IV secretion system proteins TrbI TraG and TraF, two encoded putative secreted proteins, two encoded type III secretion system proteins XopA and XopAD, two encoded DNA topoisomerases, and others encoded proteins of various functions (Table 3).

### Specificity to common bean is associated with successive waves of horizontal gene transfers

Strain CFBP6546 from the NF1 lineage was used as reference for further analyses. Its genome contained one chromosome and three extrachromosomal plasmids formerly described as plasmid a, plasmid b and plasmid c in strain 4834-R [26]. Most candidate genes (95/115) were located on the chromosome, while 20 were located on the plasmid a (Fig. 3). This corresponds to a density of one candidate gene per 50.9 kbp in the chromosome, and one per 3.5 kbp for the plasmid a, while none were retrieved in plasmids b or c. Interestingly, all the CDS found in plasmid a contained specific nonsynonymous sites (Table 3). Thus, plasmid a appeared as an important vector of genes involved in the adaptation to common bean. Another observation was that the chromosome contained regions with various 24-mers shared by the NF1 lineage and any of the fuscans, NF2 or NF3 lineages (in green, blue or black in Fig. 3, respectively). This suggests that the regions shared by the NF1 and the other lineages diverged since the split between the NF2, NF3 and fuscans lineages. By contrast to what was observed for the chromosome, all specific 24-mers found in plasmid a were simultaneously shared by the four genetic lineages of strains responsible for CBB (in purple in Fig. 3), indicating that these regions have been shared between the four lineages recently enough to still have 100% identity between each other. Together, these results suggest that 24-mers retrieved in the chromosome correspond to more ancient HGT events than those retrieved in plasmid a.

Nucleotide synonymous substitution rates at silent sites (Ks) is an estimation of neutral evolution because it does not take into account the nonsynonymous sites that can be under selection pressure. Therefore, Ks can be used as an approximation of the time of divergence between genes or taxa, with higher Ks value meaning longer time of divergence between two sequences [27, 28]. For each of the 115 candidate genes found in CBB agents, we performed multiple alignments. We could not perform Ks analysis on 15 genes that were lacking outgroups (Table 3), therefore we tested only 100 out of 115 genes. Among these 100 genes, 18 were recombinants according to RDP software analysis (Table 3). For these 18 recombinants, Ks values were independently calculated on both sides of the breakpoints. We calculated pairwise Ks values for different combinations of strains including *X. citri* pv. *fuscans*, *X. phaseoli* pv. *phaseoli* and closely related strains including *X. citri* pv. *anacardii*, *X. citri* pv. *aurantifolii*, *X. phaseoli* pv. *syngonii*, *X. phaseoli* pv. *dieffienbachiae* and *X. phaseoli* pv. *manihotis* (Fig. 1, Additional file 3). We then used these Ks as relative time divergence estimations to infer if a HGT occurred between NF1, NF2, NF3 and/or fuscans lineages, as well as the direction of this HGT. For example, for gene *m00100580b* the mean Ks value between strains from the NF1 and fuscans lineages was 7.40e-03 +/− 2.85e-10. This value was lower than the Ks values when comparing the fuscans lineage to its closest relatives from the NF2 or NF3 lineages (Ks = 4.58e-02 +/− 1.32e-09 or 4.08e-02 +/− 1.54e-09, respectively), or when comparing the NF1 lineage to its closest relative *X. phaseoli* pv. *manihotis* (Ks = 5.33e-01 +/− 0.00). These results indicate that *m00100580b* was more similar between the NF1 and fuscans lineages than between these lineages and their closest relatives, meaning that *m00100580b* was horizontally transferred between the ancestors of the NF1 and fuscans lineages. Moreover, the Ks value between the NF1 lineage and the NF2 or NF3 lineages (Ks = 4.36e-02 +/− 1.04e-09 or 4.01e-02 +/− 0.00, respectively) was lower than between the NF1 lineage and *X. phaseoli* pv. *manihotis* (Ks = 5.33e-01 +/− 0.00). Therefore, *m00100580b* was closer between NF1 strains and other strains from group 9.6 than from it’s closest relatives, meaning that the horizontal transfer was directed from the fuscans lineage to the NF1 lineage. This analysis confirmed HGT for 88 out of 100 genes tested (Fig. 1, Table 3, Additional file 3). The vast majority of HGT was directed from *X. citri* pv. *fuscans* to *X. phaseoli* pv. *phaseoli*, while only four HGT occurred from *X. phaseoli* pv. *phaseoli* to *X. citri* pv. *fuscans*. In particular, 55 HGT events were detected from the fuscans lineage to the NF1 lineage. In addition to having been transferred between distant lineages, 16 and 1 genes were also transferred between the fuscans and the NF2 lineages, or the fuscans and the NF3 lineages, respectively (Fig. 1). Moreover, eight genes had Ks = 0.00 +/− 0.00 between the NF2 and NF3 lineages, and nine between the NF2, NF3 and fuscans lineages, suggesting that HGT events also occurred between these lineages (Fig. 1, Additional file 3). Together, our results show that several more or less important waves of HGT occurred between the ancestors of phylogenetically distant strains responsible for CBB.

Finally, GC content is often used as a mean to detect HGT from foreign origin [29]. Out of the 115 candidate CDS, only two CDS (m00105390 and m00200160) presented an atypical GC content (α < 0.05) within the genome of strain CFBP6546 (Table 3). This result was not unexpected, as all strains from the NF1, NF2, NF3 and fuscans lineages have a similar GC content around 64% (Table 1), therefore HGT between these strains was not expected to result in a shift of GC content.

## Discussion

We performed a comparative genomics analysis to detect genes putatively involved in *Xanthomonas* specificity to common bean. For this, we generated the whole genome sequence from 17 strains representing the diversity of the four genetic lineages belonging to *X. citri* pv. *fuscans* and *X. phaseoli* pv. *phaseoli*. We used a combination of comparative genomics approaches that led to the discovery of 115 genes bearing features unique to CBB agents. Out of these 115 genes, 108 were retrieved using the SkIf tool based on specific 24-mer search [25]. Previous analyses based on identity percentage unveiled 63 genes sharing 100% identity over at least 95% of their length among strains from the NF1 and fuscans linages [17]. Only nine of these genes were retrieved within our list of 115 genes (Table 3). This difference can be explained by the fact that we discarded most of the genes shared only by the NF1 and fuscans lineages and retained genes similar in all four genetic lineages of CBB agents (Table 2). On the other hand, we unveiled numerous genes that did not share 100% identity among the NF1 and fuscans lineages for their whole length, but instead shared small specific sequences of 24 nucleotides or more. Whether these similarities lie within functionally important domains of the encoded proteins remains to be studied.

Ks comparisons, showed that a majority of these genes were involved in HGT between *X. citri* pv. *fuscans* and *X. phaseoli* pv. *phaseoli*. Therefore, HGT was the predominant force leading to similarities between the genomes of *X. citri* pv. *fuscans* and *X. phaseoli* pv. *phaseoli*. Finding HGT events within these genes validated our approach. In particular, SkIf was an interesting tool because in addition to being more sensitive than gene gain and loss or phylogenetic approaches, it was not based on gene alignments, thus less sensitive to annotation and/or sequencing biases. HGT events occurred at different moments of the evolution of *Xanthomonas* strains having common bean as host. The vast majority of these HGT were directed from *X. citri* pv. *fuscans* to *X. phaseoli* pv. *phaseoli*. This strongly suggest that *X. citri* pv. *fuscans* was originally pathogenic on common bean, and that *X. phaseoli* pv. *phaseoli* subsequently acquired the ability to cause CBB on bean due to successive acquisitions of novel genes and/or novel alleles coming from the three *X. citri* pv. *fuscans* lineages. This can be compared to our knowledge on the origin of the lineages and their genetic diversity. The causal agent of CBB was first isolated and identified by Smith in 1897 [30] as a yellow pigmented strain, later shown as belonging to the NF1 lineage. Burkholder later isolated the first fuscous strains from beans grown in Switzerland in 1924 [31]. However there are no data to document a putative pre-existence of one, the other, or both types of strains prior to their first identifications. The genetic diversity of the yellow and fuscous strains was revealed by various methods. Amplified or restriction fragment length polymorphism analyses [16, 32–34], amplified polymorphic DNA fragments [32, 35], pulse field gel electrophoresis [33], and multilocus sequence analysis [23] all revealed that both types of strains are more or less equivalent in terms of genetic diversity. This suggests that diversification of both lineages occurred around the same time, and thus that the ancestors of these two lineages may have coexisted. As a consequence, *X. citri* pv. *fuscans* may be the descendant of the original CBB agent that had transferred determinants useful for adaptation on common bean to the ancestor of *X. phaseoli* pv. *phaseoli*. Therefore, the ancestor of *X. phaseoli* pv. *phaseoli* appears as a recombinant that emerged as a new common bean pathogen through the acquisition of novel genes and alleles. In quarantine areas such as Europe, Turkey, Barhain, Azerbaijan and Israel, seed lots are routinely tested using a method from the International Seed Testing Association involving isolation of bacterial strains, pathogenicity tests, and specific PCR assays [36, 37]. Our results could serve for improving PCR-based monitoring of CBB agents by designing PCR primers on genes presenting sequences unique to CBB agents and potentially important for common bean specificity. Such primers could potentially detect novel HGT of these genes in strains unrelated to *X. phaseoli* pv. *phaseoli* and *X. citri* pv. *fuscans*, thus allowing to forecast new threads potentially dangerous for common bean production.

Very diverse functions were retrieved among the proteins encoded by the 115 candidate genes. Interestingly, 44 genes contained nonsynonymous polymorphisms specific for strains responsible for CBB, suggesting that they may play an important role in common bean specificity. Although 17 of these 44 genes encoded hypothetical proteins, it appears that most other genes encoded proteins involved in pathogenicity or in the interaction with the plant environment. The type IV secretion system was particularly represented with genes encoding *TraF*, *TraG* and *TraI*, and is involved in the translocation of macromolecules such as proteins important for pathogenicity, or DNA for mediating HGT [38, 39]. Thus, sharing similar proteins of the type IV secretion system could favour HGT among strains responsible for CBB. Indeed, strains from the NF1, NF2, NF3 and fuscans lineages have been found in La Réunion Island in 2000 (Table 1), indicating that sympatry exists among all these lineages, rendering further HGT events possible [18]. Three genes encoding proteins related to the type III secretion system were retrieved. XopA [40] and HpaH [41] are two proteins that may be involved in the structure of the type III secretion system, while XopAD is a type III effector of unknown function consisting of multiple semi-conserved 42 amino acids SKW repeats [42, 43]. The type III secretion system is pivotal for the virulence of most Gram negative plant pathogenic bacteria, and repertoires of type III effectors have been described as potentially important factors for host specificity and host adaptation in *Xanthomonas* [7, 44, 45] and other genera such as *Pseudomonas* [46]. Moreover, our analysis pointed out one diguanilate cyclase and one cGMP specific phosphodiesterase, two proteins involved in the metabolism of cyclic di-GMP that may play a role in biofilm formation [47] and pathogenicity [48]. One TonB-dependent transporter was also retrieved. TonB-dependent transporter are outer membrane receptors involved in molecule uptake such as iron siderophore complexes or nutrients and may play a role in host specificity [3, 49]. Other proteins putatively involved in pathogenicity were retrieved, such as ThrC, a threonine synthase involved in the virulence of *X. oryzae* pv. *oryzicola* in rice [50], XpsD, an outer membrane protein from the type II secretion system that is putatively involved in the secretion of cell wall degradative enzymes during infection [51], or IcmF, a protein of the type VI secretion system, which is involved in the interaction with other bacteria and may participate in pathogenicity [52, 53]. One flavine reductase and one xanthine dehydrogenase, two proteins putatively involved in oxydoreduction pathways were retrieved, and may be involved in the response to stress during the interaction with common bean. In addition to genes putatively involved in pathogenicity or virulence, our analysis unveiled genes involved in more general metabolism pathways, such as PhnB involved in the biosynthesis of tryptophan [54], or two DNA topoisomerases involved in the relaxation of the supercoiled DNA molecule during transcription, replication or recombination [55]. On one hand, our analysis unveiled only one or few genes within a given function, while the functions retrieved correspond to pathways often involving dozens of genes. This suggested that slight modifications within a given pathway would be sufficient to impact host specificity. On the other hand, the genes retrieved here encompass almost all the stages of host plant colonization by the bacteria, from the ability to mobilize trophic resources for multiplication to the interaction with other microorganisms, biofilm formation, response to oxidative stress, and inhibition of plant defences. Therefore, the ability to infect a particular plant seems to require not just one or a few adaptative determinants but an arsenal of factors allowing a global adaptation to a specific niche including the plant and, as a consequence a fine tuning and coordination of the activity of these determinants.

Interestingly, 19 of these 44 candidate genes were retrieved on plasmid a, suggesting that this plasmid played a major role for pathological convergence of CBB agents. Plasmid a carries an additional type III effector gene encoding an effector from the Transcription Activator-Like (TAL) family that was horizontally transferred between the NF1, NF2, NF3 and fuscans lineages [18]. Plasmids are genetic elements that favour HGT, but transfers of whole plasmids often induce a fitness cost for the bacteria [56]. More generally, horizontally transferred genes tend to be lost if not providing selective advantages for recipient strains [57]. Interestingly, the nine candidate genes retrieved by the gain and loss approach were all located on plasmid a (Table 3). The maintenance of these novel genes in the four genetic lineages of CBB agents is a testament to the importance of these genes for the bacteria. Except for two genes encoding proteins involved in the type IV secretion system, the other seven genes encoded proteins of unknown function. It would be interesting to perform functional characterization of these genes, and further analyse their implication in common bean specificity. Analysing the expression patterns during infection would be a natural extension of this study, and a first step towards functional validation of these genes.

## Conclusion

Together, our results indicate that consecutive waves of HGT occurred between phylogenetically distant *Xanthomonas* strains able to cause the same symptoms on the same host plant: common bean. These HGT led to specific combinations of genes only retrieved in strains responsible for CBB, which provided new insights into the evolution of these bacteria towards infecting common bean. Mining for candidate genes for host specificity could be generalized to other polyphyletic pathovars such as pathovars *euvesicatoria, vesicatoria, perforans,* and *gardneri* forming a group of strains pathogenic on pepper and tomato [8]. Such analyses could both give new information on the molecular bases of host specificity, and provide new tools for enhancing epidemiological surveillance of strains pathogenic on a given host, or detecting recombinant strains presenting a high potential of emergence through the acquisition of novel genes.

## Methods

### Bacterial genomes and strains

All strains used in this study are listed in Table 1. The strains used for genome sequencing were provided by the CIRM-CFBP (International Center for Microbial Ressources - French Collection for Plant-associated Bacteria, https://www6.inra.fr/cirm_eng/). Genome sequencing was performed using the following procedure. Genomic DNA was prepared from overnight liquid cultures of bacteria previously grown on 10% TSA medium (tryptone at 1.7 g/L, soybean peptone at 0.3 g/L, glucose at 0.25 g/L, NaCl at 0.5 g/L, K_2_HPO_4_ at 0.5 g/L, agar at 15 g/L, pH 7.2) for 2 days at 28 °C. DNA was extracted and purified by the method of Klotz and Zimm [58]. Illumina sequencing was performed by Genoscope (20 strains, paired end reads of 300/500 bp) or GATC Biotech (three strains, with combined paired-end reads of ca. 250 bp and 3 kb mate-pair reads). Genome assembly was performed using a combination of SOAPdenovo (version 2.04) [59], SOAPGapCloser (version 1.12) [60] and Velvet (version 1.2.02) [61] assemblers. Sequenced genomes were estimated to be > 93% complete and < 3% contaminated (Additional file 4) using CheckM (version 1.0) [62]. The pathogenicity of all CBB strains was confirmed on common bean plants from cultivar Flavert as described in Ruh et al. [18]. The seeds from cultivar Flavert were kindly provided by Vilmorin (La Ménitré, France) and are available at the bean collection of the CIAT (Center for Tropical Agriculture, Colombia, http://genebank.ciat.cgiar.org/genebank/main.do).

### Annotation and phylogenomic analyses

Structural and functional annotation of whole genome assemblies was performed using the automated pipeline Eugene-PP (version 1.2) [63], using SWISS-PROT as protein database and training protein database (http://www.uniprot.org/). Additional functional annotation of all predicted CDS was performed with InterProScan (version 4) [64]. A presence/absence matrix of ortholog groups was constructed using OrthoMCL (version 2.0) on amino acid sequences from all predicted CDS at an inflation index of 1.5 [65]. This matrix was then used for defining core and pan genomes. Phylogenetic trees were constructed using CVTree (version 4.2) [66] using the aminoacid sequences of all predicted CDS from the 75 genomes used in this study. CDS gains and losses were analysed using the Most Parcimonious Reconstruction function from the APE package (version 3.2) [67] to search for the most parsimonious succession of events explaining the repertoire of ortholog groups at each node of the phylogenetic tree.

### Searching for genes monophyletic for *X. citri* pv*. fuscans* and *X. phaseoli* pv*. phaseoli* strains

A phylogenetic approach was used to search for genes for which strains from *X. citri* pv. *fuscans* and *X. phaseoli* pv. *phaseoli* form a monophyletic group. For this we selected 3202 CDS using an *R* script to search the orthology matrix for genes that were present in all *X. citri* pv. *fuscans* and *X. phaseoli* pv. *phaseoli* strains and in at least one strain from Rep-PCR group 9.2 or 9.4 [11] plus one another strain from Rep-PCR group 9.5 or 9.6, in order to avoid getting trees were *X.citri* pv. *fuscans* and *X. phaseoli* pv. *phaseoli* appear monophyletic due to a lack of correspondig genes in the strains inbetween. CDS were aligned using MAFFT (version 7) with L-INS-I strategy [68]. Neighbour-joining trees were constructed using APE (version 3.2) under the Kimura 80 model [67]. CDS monophyletic for all *X. citri* pv. *fuscans* and *X. phaseoli* pv. *phaseoli* strains, or alternatively for the NF1 and another lineage (i.e. NF2, NF3, or fuscans), were retrieved using the APE package (version 3.2) [67].

### Searching for genes containing *k*-mers specific for *X. citri* pv*. fuscans* and *X. phaseoli* pv*. phaseoli* strains

A *k*-mer-based approach was used to search for genes containing short specific sequences present in all strains from *X. citri* pv. *fuscans* and *X. phaseoli* pv. *phaseoli* but absent in other strains. For this, we used SkIf (version 1.0) [25] with a *k*-mer size of 24 (or 24-mer), and using *X. citri* pv. *fuscans* strain 4834-R genome as reference. The same approach was used to search for genes containing 24-mers absent in strains belonging to *X. citri* pv. *fuscans* and *X. phaseoli* pv. *phaseoli* but conserved in all other strains from the *X*. *axonopodis* species complex, using *X. citri* pv. *anacardii* strain CFBP2913 genome as reference.

### Recombination and HGT analyses

The 115 genes presenting specific traits of adaptation to common bean were aligned using MAFFT (version 7) with L-INS-I strategy [68]. Intragenic recombination events were then searched using a suite of programs implemented in RDP (version 4.16) [69], RDP [70], Geneconv [71], MaxChi [72], Chimaera [73], Bootscan [74] and 3seq [75]. Default parameters were used for each method except for Bootscan (window = 150, step = 20, neighbor joining trees, 200 replicates, 95% cut-off, J&N model with Ti:Tv = 2, coefficient of variation = 2). *Ks* was calculated using DNAsp (version 5) [76]. For each gene, the occurrence and dating of HGT events were estimated by comparing *Ks* values from 28 different pairwise combinations listed in Additional file 3. For example, NF1 and fuscans strains belong to phylogenetically distant strains, thus if the *Ks* between strains from genetic lineages NF1 and fuscans was lower than the mean *Ks* between other lineages, it was indicative of recent HGT between the ancestors of NF1 and fuscans. Direction of events were assessed by comparing the *Ks* values for outgroups belonging to Rep-PCR groups 9.4 and 9.6 (Fig. 1). For recombinants, separate analyses were performed for each region on both sides of the recombination point.

## Additional files

Additional file 1: CDS content per strain. (PDF 406 kb)

Additional file 2: OrthoMCL matrix. (XLSX 10182 kb)

Additional file 3: Ks analysis on 100 CDS presenting characteristics unique to CBB agents. (XLSX 64 kb)

Additional file 4: CheckM analysis of the genomes sequenced in this study. (XLSX 11 kb)

## Abbreviations

CBB: Common bacterial blight of bean
CDS: Coding sequence
HGT: Horizontal gene transfer
*Ks*: Nucleotide synonymous substitution rate at silent sites
NF1, 2 or 3: Non-fuscous lineages 1, 2 or 3
PTI: Pattern triggered immunity (PTI)
rep-PCR: Repetitive-sequence-based Polymerase Chain Reaction
TAL: Transcription activator-like

## References

[1] Bäumler A, Fang FC. Host specificity of bacterial pathogens. Cold Spring Harb Perspect Med. 2013;3:a010041. Available from: http://perspectivesinmedicine.cshlp.org/content/3/12/a010041. 10.1101/cshperspect.a010041 PMC3839602 24296346

[2] Büttner D, Bonas U. Regulation and secretion of Xanthomonas virulence factors. FEMS Microbiol Rev. 2010;34:107–33. 10.1111/j.1574-6976.2009.00192.x 19925633

[3] Jacques M-A, Arlat M, Boulanger A, Boureau T, Carrère S, Cesbron S (2016). Using ecology, physiology, and genomics to understand host specificity in Xanthomonas: French Network on Xanthomonads (FNX). Annu Rev Phytopathol.

[4] Hayward AC (1993). The hosts of Xanthomonas. Xanthomonas.

[5] Dye DW, Bradbury JF, Goto M, Hayward AC, Lelliott RA, Schroth MN (1980). International standards for naming pathovars of phytopathogenic bacteria and a list of pathovar names and pathotype strains. Rev Plant Pathol.

[6] Mhedbi-Hajri N, Darrasse A, Pigné S, Durand K, Fouteau S, Barbe V (2011). Sensing and adhesion are adaptive functions in the plant pathogenic xanthomonads. BMC Evol Biol.

[7] Hajri A, Brin C, Hunault G, Lardeux F, Lemaire C, Manceau C, et al. A «repertoire for repertoire» hypothesis: repertoires of type three effectors are candidate determinants of host specificity in Xanthomonas. PLoS One. 2009;4:e6632. 10.1371/journal.pone.0006632 PMC2722093 19680562

[8] Schwartz AR, Potnis N, Timilsina S, Wilson M, Patané J, Martins J, et al. Phylogenomics of Xanthomonas field strains infecting pepper and tomato reveals diversity in effector repertoires and identifies determinants of host specificity. Front Microbiol. 2015;6:535. 10.3389/fmicb.2015.00535 PMC4452888 26089818

[9] Vauterin L, Hoste B, Kersters K, Swings J (1995). Reclassification of Xanthomonas. Int J Syst Bacteriol.

[10] Vauterin L, Rademaker J, Swings J (2000). Synopsis on the taxonomy of the genus Xanthomonas. Phytopathology.

[11] Rademaker JLW, Louws FJ, Schultz MH, Rossbach U, Vauterin L, Swings J, et al. A comprehensive species to strain taxonomic framework for Xanthomonas. Phytopathology. 2005;95:1098–1111. 10.1094/PHYTO-95-1098 18943308

[12] Constantin EC, Cleenwerck I, Maes M, Baeyen S, Van Malderghem C, De Vos P (2016). Genetic characterization of strains named as Xanthomonas axonopodis pv. dieffenbachiae leads to a taxonomic revision of the X. axonopodis species complex. Plant Pathol.

[13] Oren A, Garrity GM (2017). Notification of changes in taxonomic opinion previously published outside the IJSEM. Int J Syst Evol Microbiol.

[14] EFSA PLH Panel (EFSA Panel on Plant Health) (2015). Scientific opinion on the pest categorisation of Spodoptera littoralis. EFSA J.

[15] Belete T, Bastas K. Common Bacterial Blight (Xanthomonas axonopodis pv. phaseoli) of Beans with Special Focus on Ethiopian Condition. J Plant Pathol Microbiol. 2017;8:403. Available from: https://www.omicsonline.org/open-access/common-bacterial-blight-xanthomonas-axonopodis-pv-phaseoli-of-beans-with-special-focus-on-ethiopian-condition-2157-7471-1000403.php?aid=87779.

[16] Alavi SM, Sanjari S, Durand F, Brin C, Manceau C, Poussier S (2008). Assessment of the genetic diversity of Xanthomonas axonopodis pv. phaseoli and Xanthomonas fuscans subsp. fuscans as a basis to identify putative pathogenicity genes and a type III secretion system of the SPI-1 family by multiple suppression subtractive h. Appl Environ Microbiol.

[17] Aritua V, Harrison J, Sapp M, Buruchara R, Smith J, Studholme DJ (2015). Genome sequencing reveals a new lineage associated with lablab bean and genetic exchange between Xanthomonas axonopodis pv. phaseoli and Xanthomonas fuscans subsp. fuscans. Front Microbiol.

[18] Ruh M, Briand M, Bonneau S, Jacques M-A, Chen NWG (2017). Xanthomonas adaptation to common bean is associated with horizontal transfers of genes encoding TAL effectors. BMC Genomics.

[19] Simpson AJG, Reinach FC, Arruda P, Abreu FA, Acencio M, Alvarenga R (2000). The genome sequence of the plant pathogen Xylella fastidiosa. Nature.

[20] van Sluys MA, de Oliveira MC, Miyaki CY, Furlan LR, Camargo LEA, Silva ACR, et al. Comparative analyses of the complete genome sequences of Pierce’s disease and citrus variegated chlorosis strains of Xylella fastidiosa. J Bacteriol. 2003;185:1018–26. 10.1128/JB.185.3.1018-1026.2003 PMC142809 12533478

[21] Taghavi S, Garafola C, Monchy S, Newman L, Hoffman A, Weyens N, Barac T, et al. Genome survey and characterization of endophytic bacteria exhibiting a beneficial effect on growth and development of poplar trees. Appl Environ Microbiol. 2009;75:748–57. 10.1128/AEM.02239-08 PMC2632133 19060168

[22] Jacques M-A, Arlat M, Boulanger A, Boureau T, Carrère S, Cesbron S (2016). Using ecology, physiology, and genomics to understand host specificity in *Xanthomonas*. Annu Rev Phytopathol.

[23] Mhedbi-Hajri N, Hajri A, Boureau T, Darrasse A, Durand K, Brin C, et al. Evolutionary history of the plant pathogenic bacterium Xanthomonas axonopodis. PLoS One. 2013;8:e58474. 10.1371/journal.pone.0058474 PMC3591321 23505513

[24] Young JM, Park DC, Shearman HM, Fargier E (2008). A multilocus sequence analysis of the genus Xanthomonas. Syst Appl Microbiol.

[25] Briand M, Gaborieau R, Jacques M-A, Barret M, Boureau T, Gaillard S (2016). SkIf: a tool for rapid identification of genes or regulators of interest [version 1; not peer reviewed]. F1000Res.

[26] Darrasse A, Bolot S, Serres-Giardi L, Charbit E, Boureau T, Saux MF-L (2013). High-quality draft genome sequences of Xanthomonas axonopodis pv. glycines strains CFBP 2526 and CFBP 7119. Genome Announc.

[27] Ochman H, Elwyn S, Moran NA. Calibrating bacterial evolution. Proc Natl Acad Sci U S A. 1999;96:12638–12643. Available from: http://www.pnas.org/content/96/22/12638. 10.1073/pnas.96.22.12638 PMC23026 10535975

[28] Kuo C-H, Ochman H (2009). Inferring clocks when lacking rocks: the variable rates of molecular evolution in bacteria. Biol Direct.

[29] Ravenhall M, Nives Š, Lassalle F, Dessimoz C (2015). Inferring horizontal gene transfer.

[30] Zaumeyer WJ. The bacterial blight of beans caused by Bacterium phaseoli. Washington: United States Department of Agriculture; 1930.

[31] Burkholder WH (1930). The bacterial diseases of the bean: a comparative study.

[32] Birch PRJ, Hyman LJ, Taylor R, Opio AF, Bragard C, Toth IK (1997). RAPD PCR-based differentiation of Xanthomonas campestris pv. Phaseoli and Xanthomonas campestris pv. phaseoli var. fuscans. Eur J Plant Pathol.

[33] Chan JWYF, Goodwin PH (1999). Differentiation of Xanthomonas campestris pv. phaseoli from Xanthomonas campestris pv. phaseoli var. fuscans by PFGE and RFLP. Eur J Plant Pathol.

[34] Lazo GR, Gabriel DW. Conservation of plasmid DNA sequences and pathovar identification of strains of Xanthomonas campestris. Phytopathology. 1987;77:448–53.

[35] Mkandawire ABC, Mabagala RB, Guzmán P, Gepts P, Gilbertson RL. Genetic diversity and pathogenic variation of common blight bacteria (Xanthomonas campestris pv. phaseoli and X. campestris pv. phaseoli var. fuscans) suggests pathogen coevolution with the common bean. Phytopathology. 2004;94:593–603. 10.1094/PHYTO.2004.94.6.593 18943484

[36] Audy P, Laroche A, Saindon G, Huang HC, Gilbertson RL. Detection of the bean common blight bacteria, Xanthomonas campestris pv. phaseoli and X. c. phaseoli var. fuscans, using the polymerase chain reaction. Mol Plant Pathol. 1994;84:1185–92.

[37] Grimault V, Olivier V, Rolland M, Darrasse A, Jacques MA. Seed health testing methods. 7-021: Detection of Xanthomonas axonopodis pv. phaseoli on Phaseolus vulgaris. ISTA Int rules seed testing Annex to Chapter 7 Seed Heal methods 7-021 Int Seed Test Assoc Basserdorf, Switzerland, 1–20. 2014.

[38] Fronzes R, Christie PJ, Waksmas G (2009). The structural biology of type IV secretion systems. Nat Rev Microbiol.

[39] Wallden K, Rivera-Calzada A, Waksman G (2010). Type IV secretion systems: versatility and diversity in function. Cell Microbiol.

[40] Noël L, Thieme F, Nennstiel D, Bonas U (2002). Two novel type III-secreted proteins of Xanthomonas campestris pv. vesicatoria are encoded within the hrp pathogenicity island. J Bacteriol.

[41] Hausner J, Hartmann N, Jordan M, Büttner D. The predicted lytic transglycosylase HpaH from xanthomonas campestris pv. vesicatoria associates with the Type III secretion system and promotes effector protein translocation. Infect Immun. 2017;85:1–18. 10.1128/IAI.00788-16 PMC5278175 27895129

[42] Teper D, Burstein D, Salomon D, Gershovitz M, Pupko T, Sessa G (2016). Identification of novel Xanthomonas euvesicatoria type III effector proteins by a machine-learning approach. Mol Plant Pathol.

[43] Escalon A, Javegny S, Vernière C, Noël LD, Vital K, Poussier S (2013). Variations in type III effector repertoires, pathological phenotypes and host range of Xanthomonas citri pv. citri pathotypes. Mol Plant Pathol.

[44] Hersemann L, Wibberg D, Blom J, Goesmann A, Widmer F, Vorholter F-J (2017). Comparative genomics of host adaptive traits in Xanthomonas translucens pv. graminis. BMC Genomics.

[45] Barak JD, Vancheva T, Lefeuvre P, Jones JB, Timilsina S, Minsavage GV (2016). Whole-genome sequences of *Xanthomonas euvesicatoria* strains clarify taxonomy and reveal a stepwise erosion of type 3 effectors. Front Plant Sci.

[46] Baltrus DA, Nishimura MT, Romanchuk A, Chang JH, Mukhtar MS, Cherkis K, et al. Dynamic evolution of pathogenicity revealed by sequencing and comparative genomics of 19 pseudomonas syringae isolates. PLoS Pathog. 2011;7:e1002132. 10.1371/journal.ppat.1002132 PMC3136466 21799664

[47] Newell PD, Boyd CD, Sondermann H, O’Toole GA (2011). A c-di-GMP effector system controls cell adhesion by inside-out signaling and surface protein cleavage. PLoS Biol.

[48] Su J, Zou X, Huang L, Bai T, Liu S, Yuan M (2016). DgcA, a diguanylate cyclase from Xanthomonas oryzae pv. oryzae regulates bacterial pathogenicity on rice. Sci Rep.

[49] Blanvillain S, Meyer D, Boulanger A, Lautier M, Guynet C, Denancé N, et al. Plant carbohydrate scavenging through TonB-dependent receptors: a feature shared by phytopathogenic and aquatic bacteria. PLoS One. 2007;2:e224. 10.1371/journal.pone.0000224 PMC1790865 17311090

[50] Guo W, Cui YP, Li YR, Che YZ, Yuan L, Zou LF (2012). Identification of seven Xanthomonas oryzae pv. oryzicola genes potentially involved in pathogenesis in rice. Microbiology.

[51] Solé M, Scheibner F, Hoffmeister AK, Hartmann N, Hause G, Rother A (2015). Xanthomonas campestris pv. vesicatoria secretes proteases and xylanases via the Xps type II secretion system and outer membrane vesicles. J Bacteriol.

[52] Records AR, Gross DC (2010). Sensor kinases RetS and LadS regulate Pseudomonas syringae type VI secretion and virulence factors. J Bacteriol.

[53] Alteri CJ, Mobley HLT. The versatile type VI secretion system. Microbiol Spectr. 2016;4:1–26. 10.1128/microbiolspec.VMBF-0026-2015 PMC4887148 27227310

[54] Xie G, Bonner CA, Brettin T, Gottardo R, Keyhani NO, Jensen RA (2003). Lateral gene transfer and ancient paralogy of operons containing redundant copies of tryptophan-pathway genes in Xylella species and in heterocystous cyanobacteria. Genome Biol.

[55] Hashimi H, Zíková A, Panigrahi AK, Stuart KD, Lukeš J (2008). TbRGG1, an essential protein involved in kinetoplastid RNA metabolism that is associated with a novel multiprotein complex. RNA.

[56] Soucy SM, Huang J, Gogarten JP (2015). Horizontal gene transfer: building the web of life. Nat Rev Genet.

[57] Koskiniemi S, Sun S, Berg OG, Andersson DI (2012). Selection-driven gene loss in bacteria. PLoS Genet.

[58] Klotz LC, Zimm BH (1972). Size of DNA determined by viscoelastic measurements: results on bacteriophages, Bacillus subtilis and Escherichia coti. J Mol Biol.

[59] Li R, Zhu H, Ruan J, Qian W, Fang X, Shi Z (2010). De novo assembly of human genomes with massively parallel short read sequencing. Genome Res.

[60] Luo R, Liu B, Xie Y, Li Z, Huang W, Yuan J (2012). SOAPdenovo2: an empirically improved memory-efficient short-read de novo assembler. Gigascience.

[61] Zerbino DR, Birney E (2008). Velvet: algorithms for de novo short read assembly using de Bruijn graphs. Genome Res.

[62] Parks DH, Imelfort M, Skennerton CT, Hugenholtz P, Tyson GW. CheckM: assessing the quality of microbial genomes recovered from isolates, single cells, and metagenomes. 2015;25:1043–55. 10.1101/gr.186072.114 PMC4484387 25977477

[63] Sallet E, Gouzy J, Schiex T (2014). EuGene-PP: a next-generation automated annotation pipeline for prokaryotic genomes. Bioinformatics.

[64] Quevillon E, Silventoinen V, Pillai S, Harte N, Mulder N, Apweiler R (2005). InterProScan: protein domains identifier. Nucleic Acids Res.

[65] Li L, Stoeckert CJ, Roos DS (2003). OrthoMCL: identification of ortholog groups for eukaryotic genomes. Genome Res.

[66] Qi J, Luo H, Hao B (2004). CVTree: a phylogenetic tree reconstruction tool based on whole genomes. Nucleic Acids Res.

[67] Paradis E, Claude J, Strimmer K (2004). APE: analyses of phylogenetics and evolution in R language. Bioinformatics.

[68] Katoh K, Kuma KI, Toh H, Miyata T (2005). MAFFT version 5: Improvement in accuracy of multiple sequence alignment. Nucleic Acids Res.

[69] Martin DP, Murrell B, Golden M, Khoosal A, Muhire B (2015). RDP4: detection and analysis of recombination patterns in virus genomes. Virus Evol.

[70] Martin D, Rybicki E (2000). RDP: detection of recombination amongst aligned sequences. Bioinformatics.

[71] Padidam M, Sawyer S, Fauquet CM (1999). Possible emergence of new geminiviruses by frequent recombination. Virology.

[72] Smith JM (1992). Analyzing the mosaic structure of genes. J Mol Evol.

[73] Posada D, Crandall KA (2001). Evaluation of methods for detecting recombination from DNA sequences: computer simulations. Proc Natl Acad Sci.

[74] Martin DP, Posada D, Crandall KA, Williamson C (2005). A modified bootscan algorithm for automated identification of recombinant sequences and recombination breakpoints. AIDS Res Hum Retrovir.

[75] Boni MF, Posada D, Feldman MW (2007). An exact nonparametric method for inferring mosaic structure in sequence triplets. Genetics.

[76] Librado P, Rozas J (2009). DnaSP v5: a software for comprehensive analysis of DNA polymorphism data. Bioinformatics.

[77] Pieretti I, Royer M, Barbe V, Carrere S, Koebnik R, Cociancich S (2009). The complete genome sequence of Xanthomonas albilineans provides new insights into the reductive genome evolution of the xylem-limited Xanthomonadaceae. BMC Genomics..

[78] Roux B, Bolot S, Guy E, Denancé N, Lautier M, Jardinaud M-F, et al. Genomics and transcriptomics of Xanthomonas campestris species challenge the concept of core type III effectome. BMC Genomics [Internet]. BMC Genomics. 2015;16:975. Available from: https://bmcgenomics.biomedcentral.com/articles/10.1186/s12864-015-2190-0. 10.1186/s12864-015-2190-0 PMC4652430 26581393

[79] Qian W, Jia Y, Ren S, He Y, Feng J, Lu L, et al. Comparative and functional genomic analyses of the pathogenicity of phytopathogen Xanthomonas campestris pv. campestris. Genome Res. 2005;757–67. 10.1101/gr.3378705 PMC1142466 15899963

[80] da Silva ACR, Ferro JA, Reinach FC, Farah CS, Furlan LR, Quaggio RB (2002). Comparison of the genomes of two Xanthomonas pathogens with differing host specificities. Nature..

[81] Vorhölter F-J, Schneiker S, Goesmann A, Krause L, Bekel T, Kaiser O, et al. The genome of Xanthomonas campestris pv. campestris B100 and its use for the reconstruction of metabolic pathways involved in xanthan biosynthesis. J Biotechnol. 2008;134:33–45. 10.1016/j.jbiotec.2007.12.013 18304669

[82] Studholme DJ, Kemen E, MacLean D, Schornack S, Aritua V, Thwaites R (2018). Genome-wide sequencing data reveals virulence factors implicated in banana Xanthomonas wilt. FEMS Microbiol Lett..

[83] Bogdanove AJ, Koebnik R, Lu H, Furutani A, Angiuoli SV, Patil PB (2011). Two New Complete Genome Sequences Offer Insight into Host and Tissue Specificity of Plant Pathogenic Xanthomonas spp. J Bacteriol.

[84] Bolot S, Guy E, Carrere S, Barbe V, Arlat M, Noël LD. Genome Sequence of Xanthomonas campestris pv. campestris Strain Xca5. Genome Announc [Internet]. 2013;1:32–3. Available from: http://genomea.asm.org/cgi/doi/10.1128/genomeA.00032-12. 10.1128/genomeA.00032-12 PMC3569304 23405315

[85] Moreira LM, Almeida NF, Potnis N, Digiampietri LA, Adi SS, Bortolossi JC, et al. Novel insights into the genomic basis of citrus canker based on the genome sequences of two strains of Xanthomonas fuscans subsp. aurantifolii. BMC Genomics. 2010;11. 10.1186/1471-2164-11-238 PMC2883993 20388224

[86] Gordon JL, Lefeuvre P, Escalon A, Barbe V, Cruveiller S, Gagnevin L, et al. Comparative genomics of 43 strains of Xanthomonas citri pv. citri reveals the evolutionary events giving rise to pathotypes with different host ranges. BMC Genomics [Internet]. BMC Genomics; 2015;16. Available from: 10.1186/s12864-015-2310-x. 10.1186/s12864-015-2310-x PMC4690215 26699528

[87] Cunnac S, Bolot S, Serna F, Ortiz E, Szurek B, Noël LD (2013). High-Quality Draft Genome Sequences of Two Xanthomonas citri pv. malvacearum Strains. Genome Announc.

[88] Midha S, Ranjan M, Sharma V, Pinnaka AK, Patil PB. Genome sequence of xanthomonas citri pv. Mangiferaeindicae strain LMG 941. J Bacteriol. 2012;194:3031. 10.1128/JB.00433-12 PMC3370635 22582385

[89] Gagnevin L, Bolot S, Gordon JL, Pruvost O, Vernière C, Robène I, et al. Draft Genome Sequence of Xanthomonas axonopodis pv. allii Strain CFBP 6369. Genome Announc [Internet]. 2014;2:e00727-14. Available from: http://genomea.asm.org/content/2/4/e00727-14.full. 10.1128/genomeA.00727-14 PMC4118059 25081256

[90] Jalan N, Aritua V, Kumar D, Yu F, Jones JB, Graham JH (2011). Comparative genomic analysis of Xanthomonas axonopodis pv. citrumelo F1, which causes citrus bacterial spot disease, and related strains provides insights into virulence and host specificity. J Bacteriol.

[91] Thieme F, Koebnik R, Bekel T, Berger C, Boch J, Büttner D (2005). Insights into Genome Plasticity and Pathogenicity of the Plant Pathogenic Bacterium Xanthomonas campestris pv. vesicatoria Revealed by the Complete Genome Sequence Insights into Genome Plasticity and Pathogenicity of the Plant Pathogenic Bacterium. J Bacteriol.

[92] Potnis N, Krasileva K, Chow V, Almeida NF, Patil PB, Ryan RP, et al. Comparative genomics reveals diversity among xanthomonads infecting tomato and pepper. BMC Genomics [Internet]. BioMed Central Ltd; 2011;12:146. Available from: http://www.biomedcentral.com/1471-2164/12/146. 10.1186/1471-2164-12-146 PMC3071791 21396108

[93] Kimbrel JA, Givan SA, Temple TN, Johnson KB, Chang JH (2011). Genome sequencing and comparative analysis of the carrot bacterial blight pathogen, Xanthomonas hortorum pv. carotae M081, for insights into pathogenicity and applications in molecular diagnostics. Mol Plant Pathol.

[94] Lee BM, Park YJ, Park DS, Kang HW, Kim JG, Song ES (2005). The genome sequence of Xanthomonas oryzae pathovar oryzae KACC10331, the bacterial blight pathogen of rice. Nucleic Acids Res..

[95] Salzberg SL, Sommer DD, Schatz MC, Phillippy AM, Rabinowicz PD, Tsuge S (2008). Genome sequence and rapid evolution of the rice pathogen Xanthomonas oryzae pv. oryzae PXO99A. BMC Genomics.

[96] Robène I, Bolot S, Pruvost O, Arlat M, Noël LD, Carrère S (2016). High-Quality Draft Genome Sequences of Two Xanthomonas Pathotype Strains Infecting Aroid Plants..

[97] Rodriguez-R LM, Grajales A, Arrieta-Ortiz ML, Salazar C, Restrepo S, Bernal A. Genomes-based phylogeny of the genus Xanthomonas. BMC Microbiol [Internet]. BioMed Central Ltd; 2012;12:43. Available from: https://bmcmicrobiol.biomedcentral.com/articles/10.1186/1471-2180-12-43. 10.1186/1471-2180-12-43 PMC3359215 22443110

[98] Studholme DJ, Wasukira A, Paszkiewicz K, Aritua V, Thwaites R, Smith J, et al. Draft genome sequences of xanthomonas sacchari and two banana-associated xanthomonads reveal insights into the xanthomonas group 1 clade. Genes (Basel). 2011;2:1050–65. 10.3390/genes2041050 PMC3927605 24710305

[99] Pesce C, Bolot S, Cunnac S, Portier P, Fisher-Le Saux M, Jacques M-A (2015). High-Quality Draft Genome Sequence of the Xanthomonas translucens pv. cerealis Pathotype Strain CFBP 2541. Genome Announc.

[100] Pesce C, Bolot S, Berthelot E, Bragard C, Cunnac S, Fischer-Le Saux M, et al. Draft Genome Sequence of Xanthomonas translucens pv. graminis Pathotype Strain CFBP 2053. Genome Announc [Internet]. 2015b;3:15–7. Available from: https://mra.asm.org/content/3/5/e01174-15. 10.1128/genomeA.01174-15 PMC4599099 26450740

[101] Wichmann F, Vorhölter FJ, Hersemann L, Widmer F, Blom J, Niehaus K (2013). The noncanonical type III secretion system of Xanthomonas translucens pv. graminis is essential for forage grass infection. Mol Plant Pathol.

[102] van Sluys MA, de Oliveira MC, Monteiro-Vitorello CB, Miyaki CY, Furlan LR, Camargo LEA, et al. Comparative Analyses of the Complete Genome Sequences of Pierce ’ s Disease and Citrus Variegated Chlorosis Strains of Xylella fastidiosa. J Bacteriol [Internet]. 2003;185:1018–26. Available from: http://jb.asm.org/cgi/doi/10.1128/JB.185.3.1018-1026.2003. 10.1128/JB.185.3.1018-1026.2003 PMC142809 12533478

[103] Rademaker JLW, Hoste B, Louws FJ, Kersters K, Swings J, Vauterin L (2000). Comparison of AFLP and rep-PCR genomic fingerprinting with DNA – DNA homology studies: Xanthomonas as a model system. Int J Syst Evol Microbiol.

