# Supplementary figures and images for: Horizontal gene transfer plays a major role in the pathological convergence of Xanthomonas lineages on common bean
Source: BMC Genomics. 2018 Aug 13;19:606. doi: 10.1186/s12864-018-4975-4 (PMC6090828; doi:10.1186/s12864-018-4975-4)

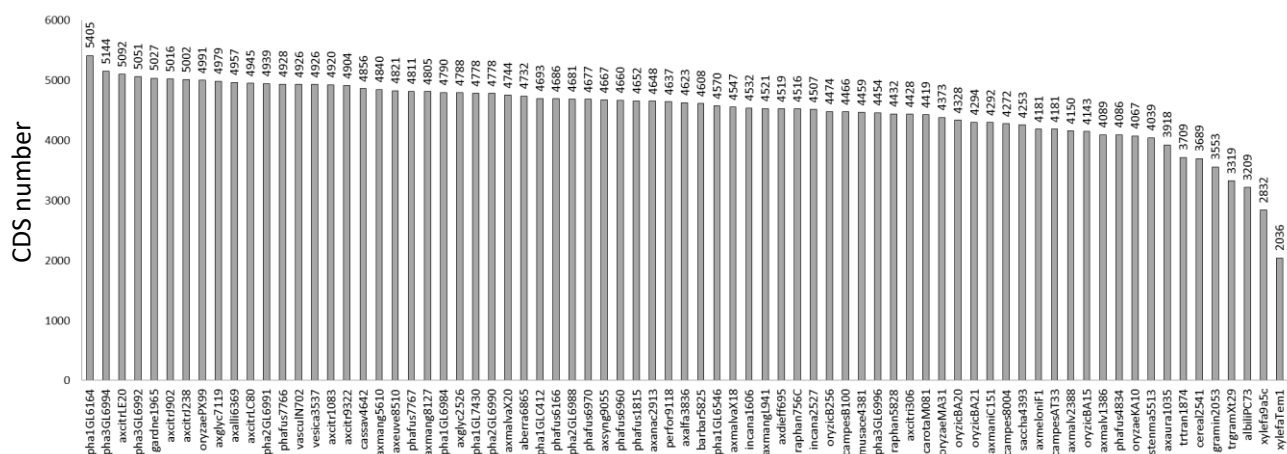

**Additional file 1:** CDS content per strain

Supplement: Supplementary file 1 — CDS content per strain. (PDF 406 kb) [file 12864_2018_4975_MOESM1_ESM.pdf]
